# Ibrutinib treatment inhibits breast cancer progression and metastasis by inducing conversion of myeloid-derived suppressor cells to dendritic cells

**DOI:** 10.1038/s41416-020-0743-8

**Published:** 2020-02-06

**Authors:** Sanjay Varikuti, Bhawana Singh, Greta Volpedo, Dinesh K. Ahirwar, Bijay K. Jha, Noushin Saljoughian, Agostinho G. Viana, Chaitenya Verma, Omar Hamza, Gregory Halsey, Erin A. Holcomb, Ritvik J. Maryala, Steve Oghumu, Ramesh K. Ganju, Abhay R. Satoskar

**Affiliations:** 1 Department of Pathology, The Ohio State University Medical Center, Columbus, OH USA; 2 Department of Microbiology, The Ohio State University, Columbus, OH USA; 3 Department of Infection and Immunity, The Ohio State University, Columbus, OH USA

**Keywords:** Cancer, Oncology

## Abstract

**Background:**

Ibrutinib is a Bruton’s tyrosine kinase (BTK) and interleukin-2-inducible kinase (ITK) inhibitor used for treating chronic lymphocytic leukaemia (CLL) and other cancers. Although ibrutinib is known to inhibit the growth of breast cancer cell growth in vitro, its impact on the treatment and metastasis of breast cancer is unclear.

**Methods:**

Using an orthotopic mouse breast cancer model, we show that ibrutinib inhibits the progression and metastasis of breast cancer.

**Results:**

Ibrutinib inhibited proliferation of cancer cells in vitro, and Ibrutinib-treated mice displayed significantly lower tumour burdens and metastasis compared to controls. Furthermore, the spleens and tumours from Ibrutinib-treated mice contained more mature DCs and lower numbers of myeloid-derived suppressor cells (MDSCs), which promote disease progression and are linked to poor prognosis. We also confirmed that ex vivo treatment of MDSCs with ibrutinib switched their phenotype to mature DCs and significantly enhanced MHCII expression. Further, ibrutinib treatment promoted T cell proliferation and effector functions leading to the induction of antitumour T_H_1 and CTL immune responses.

**Conclusions:**

Ibrutinib inhibits tumour development and metastasis in breast cancer by promoting the development of mature DCs from MDSCs and hence could be a novel therapeutic agent for the treatment of breast cancer.

## Background

Ibrutinib was primarily developed as a Bruton’s tyrosine kinase (BTK) inhibitor but was discovered to also target the inducible tyrosine kinase (ITK) and the epithelial growth factor receptor.^1,2^ Ibrutinib is highly effective in the treatment of chronic lymphocytic leukaemia (CLL), mantle cell lymphoma, and Waldenstrom’s macroglobulinaemia.^3^ Beyond its role in B cell biology, BTK functions have been explored in the maturation, trafficking, and function of myeloid cells,^4–6^ T cells,^1^ and natural killer cells.^7^ It is also shown that inhibition of ITK by ibrutinib impedes the development of T helper type 2 (T_H_2) cells and promotes T_H_1 responses.^1^ Rapid binding and high selectivity of ibrutinib reduce the risk of sustained systemic exposures, therefore making it the drug of choice with a well-tolerated dosing regimen as compared to current therapeutic options for the above diseases.

Dendritic cells (DCs) play a critical role in the induction of antitumour immunity.^8^ Normally, DCs are in an immature state, and upon exposure to external stimuli, they can undergo maturation, leading to induction of immune response against tumour antigens. Although tumour-derived factors can induce DC precursors to migrate to the tumour microenvironment,^9^ their presence does not necessarily induce antitumour responses. Furthermore, cancer cell-induced immunosuppressive microenvironment limits the activity of mature and functionally competent DCs while triggering the accumulation of tumour-promoting immature DC phenotypes.^10^ These immature DCs induce immune tolerance by the expansion of suppressor T cell populations, which regulate or suppress other immune T cells.^11^

Myeloid-derived suppressor cells (MDSCs) are a subset of immature myeloid cells that possess immunosuppressive properties. These cells expand in response to tumours, contribute to immunosuppression, and have been reported to play a role in tumour progression.^12,13^ Together, immature DCs and MDSCs can facilitate cancer progression by stimulating the development of immunosuppressive T_H_2 cells and regulatory T cells and inhibiting antitumour cytotoxic T lymphocytes (CTL) and T_H_1 cells.^9,14^

Previous studies have shown that DCs from BTK-deficient mice present a more mature phenotype, characterised by the expression of higher levels of activation markers and enhanced T cell stimulatory abilities in vitro and in vivo.^15^ Furthermore, our group has demonstrated that ibrutinib promotes DC activation and maturation, as well as T cell proliferation and augmented production of interferon (IFN)-γ.^16,17^ These findings suggest that ibrutinib could be effective in DC-based cancer therapeutics. We therefore hypothesised that ibrutinib could reprogram MDSCs to mature DCs even in the presence of a tumour-suppressive microenvironment resulting in inhibition of tumour growth and metastasis.

Recent DC-based cancer immunotherapies have focused on enhancing the proportions of mature DCs to trigger anticancer CTL responses. Our present study is based on DC-mediated anticancer therapy using the potent ITK/BTK inhibitor ibrutinib in a murine model of breast cancer. Our results show that ibrutinib decreases tumour growth and metastasis of breast cancer. Our findings also show that ibrutinib is able to reprogram MDSCs to mature DCs, which boosts antitumour T_H_1 and CTL immune responses due to improved tumour-derived antigen presentation to the T cells. Collectively, these findings indicate that ibrutinib could be a novel drug for the treatment of breast cancer.

## Methods

### Mice and tumour injections

Eight-week-old female wild-type (WT) C57BL/6 mice were purchased from Envigo (Indianapolis, IN). All experimental mice were injected with 0.1 × 10^6^ E0.2 (subclone of E0771 developed in Dr. Ramesh Ganju’s laboratory) tumour cells in 50% Matrigel (Corning, MA) into the right mammary fat pad.

### Ibrutinib treatments

Once the tumours were palpable (approximately at day 7 of tumour implantation), mice were randomised into 2 groups (*n* = 10–12 mice per group) and treated with 6 mg/Kg/day Ibrutinib (provided by Pharmacyclics LLC, an AbbVie Company) dissolved in 0.5 % methylcellulose+1% sodium lauryl sulphate (vehicle) or only vehicle from day 7 to day 30 by oral gavage.

### Tumour volume and lung metastasis counts

Tumour size was measured once every 3–4 days using a calliper, and tumour volume was calculated by using the formula: Volume = 0.52 × length × width^2^. Mice were euthanised by CO_2_ asphyxiation procedure at day 30 in compliance with OSU-IACUC. Tissues were harvested and lung metastasis was calculated by counting visible nodules.

### Cell viability and immunoblot assays

E0.2 cells were plated at 2500cells/well for overnight and treated with ibrutinib at 1, 0.5, 0.1, and 0.05 µM concentrations for 24 h. Cell viability was analysed by MTT (3-[4,5-dimethylthiazol-2-yl]-2,5 diphenyl tetrazolium bromide) cell proliferation assay (Cayman chemicals, Ann Arbor, MI). E0.2 cells were plated at 10^5^cells/well for overnight and treated with ibrutinib at 1, 0.5, 0.1, and 0.05 µM concentrations for 24 h. Cells were lysed with Pierce RIPA buffer (Thermo Scientific, Waltham, MA), and protein concentrations were measured by Pierce BCA Protein Assay Kit (Thermo Scientific, Waltham, MA). Proteins were probed for total BTK (Sigma Aldrich, St Louis, MO) and glyceraldehyde 3-phosphate dehydrogenase (Cell Signaling, Denvers, MA). Followed by the horseradish peroxidase-conjugated secondary antibody (Cell Signaling, Denvers, MA). Luminol reagent was used to develop the blot by chemiluminescence.

### Flow cytometric analysis

Mice were euthanised at the end of the study; single-cell suspensions were prepared from the spleens and tumours as described in our previous study.^18^ Spleen cells and tumour cells were stained with respective stain cocktails. Anti-mouse CD11b-PE (phycoerythrin) (101208), Ly6C-APC (allophycocyanin) (128015), Ly6G-FITC (fluorescein isothiocyanate) (127605), Gr1-PE/Cy7 (108416), CD11C-Pacific Blue (117321), major histocompatibility complex II (MHCII)-AF700 (107622), CD3-PerCP/Cy5.5 (100217), CD4-PE/Cy5.5 (100410), CD8-BV510 (100751), interleukin (IL)-2-PE (503808), IFN-γ-APC (505809), tumour necrosis factor (TNF)-α-FITC (506304), and Granzyme B-PE/Cy7 (372213) were purchased from Bio Legend (San Diego, CA). Cells were acquired through BD LSRII flow cytometer (BD Biosciences, San Jose, CA, USA). Flow cytometric analysis was performed by using the Flow Jo software (Tree Star Inc., Ashland, OR, USA). For co-cultures experiments, cells were sorted by using BD FACS ARIA III (BD Biosciences, San Jose, CA, USA).

### MDSC to DC maturation studies

WT C57BL/6 mice were injected with 0.1 × 10^6^ E0.2 (a subclone of E0771 tumour cells) in 50% matrigel (Corning, MA) into the right mammary fat pad. Once the mice developed tumours, the spleens were harvested, and single-cell suspensions were prepared. Cells were stained with CD11b, Gr1+, and total MDSCs (CD11b+Gr1+) were isolated from the spleens of tumour-bearing mice by fluorescence-activated cell sorter. MDSCs were treated with 1 µM ibrutinib or dimethyl sulfoxide (DMSO) for 1 h and then washed twice with RPMI media. Ibrutinib/DMSO treated  cells were stained with CD11C and MHCII and analysed for matured DC populations as CD11C+MHCII+ cells.

### Reverse transcription (RT)-PCR and gene expression analysis

Spleen and tumour samples were homogenised; total RNA was extracted by TRIzol extraction method (purchased from Life Technologies, Carlsbad, CA). iScript reverse transcriptase and IQ SYBR green supermix and CFX 96 RT-PCR thermocycler were used to prepare cDNA and perform RT-PCR reactions (purchased from Bio-Rad, Hercules, CA, USA). All the primers were designed according to the Harvard primer bank website (http://pga.mgh.harvard.edu/primerbank), purchased from IDT Technologies (Coralville, IA, USA). Data are represented as fold induction over WT naive mouse and normalised by using the β-actin housekeeping gene.

### Enzyme-linked immunosorbent assay (ELISA) and T cell proliferation

Cells isolated from the spleens were plated at 5 × 10^6^/ml concentration in 96-well plates and were stimulated with/without 2 µg/ml of LEAF purified anti-mouse CD3e (purchased from Biolegend) for 72 h in the complete RPMI media. Supernatants were collected and the levels of IFN-γ, TNF-α, IL-4, IL-13, IL-6, and IL-17 were quantified by ELISA (all reagents purchased from Biolegend). Cell proliferation was measured by Alamar Blue reduction technique (Bio-Rad, AbD Serotec Inc., Raleigh, NC) as described previously.^18^

### Statistical analysis

All animal studies were conducted using 10–12 animals per group for each experiment. The statistical significance was determined by using Student’s *t* test. Data represented are one of the three independent experiments.

## Results

### Ibrutinib treatment reduces breast tumour progression and tumour weight

**Fig. 1:**
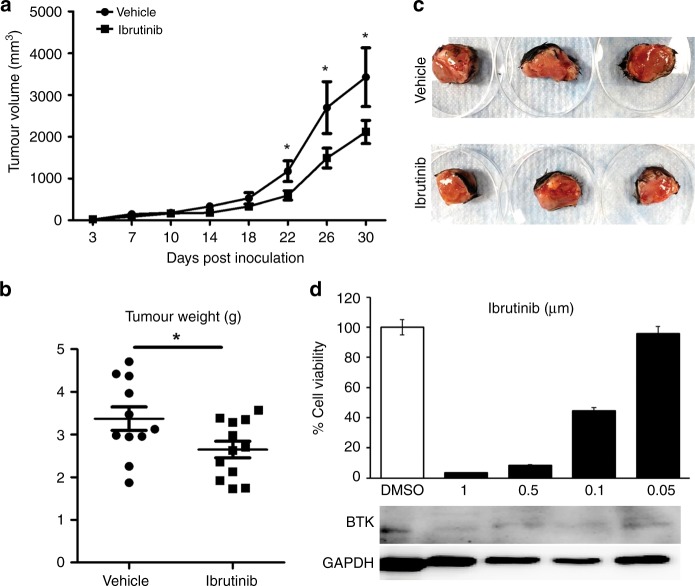
Ibrutinib reduces tumour growth and tumour size. Tumour cells were implanted into the mammary fat pad. Once the tumours were palpable, mice were randomised and treated with vehicle or ibrutinib once a day. Mice were euthanised at 30 days of post-implantation and harvested for tumours. **a** Tumour volume measured every 4 days (mean ± SE of tumour volume at each time point) are represented. **b** Tumour weights are represented in grams at day 30. **c** Representative pictures of tumours from vehicle and ibrutinib group at day 30 of harvest. **d** Percentage cell viability and immunoblot of BTK expression in E0.2 cells by ibrutinib treatment. Data represented are means ± SEM from 1 of the 3 successful experiments with a minimum of 10–12 mice per each group. *P < 0.05 by unpaired *t* test.

After implantation of tumour cells into the mammary fat pad, mice were monitored for the establishment of tumours. Once the tumours were palpable, mice were randomised into two groups and treated with vehicle or ibrutinib once a day through oral gavage. Tumour measurements were obtained every 3–4 days throughout the course of treatment. At day 30 posttreatment, mice were euthanised and tumour weights were measured. Ibrutinib-treated mice displayed a reduced tumour growth compared to controls starting from 2 weeks of posttreatment until euthanasia (Fig. 1a). In addition, tumour weights in ibrutinib-treated mice were significantly lower compared to the vehicle-treated mice (Fig. 1b, c). Next, we examined whether the effect of ibrutinib in reducing the breast tumour progression in vivo is due to the direct effect of ibrutinib on E0.2 cells. Our data show that ibrutinib treatment resulted in the viability of E0.2 tumour cells and decreased the BTK expression in a dose-dependent manner (Fig. 1d). These data indicate that ibrutinib treatment significantly reduces breast tumour growth in the murine model of breast cancer.

### Ibrutinib-treated mice show a significant reduction in tumour metastasis to the lungs

**Fig. 2:**
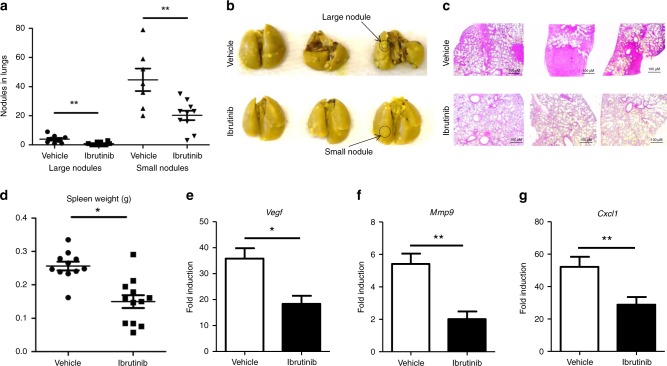
Ibrutinib treatment results in the reduction of lung metastasis. Mice were euthanised from both vehicle- and ibrutinib-treated groups at day 30, and the lungs, spleens, and tumours were collected. Lungs were placed in Bouin’s solution and analysed for both large and small metastatic nodules. **a** The numbers of large and small metastatic nodules present on the lungs of the vehicle- and ibrutinib-treated mice are represented. **b** Representative lung pictures from the vehicle- and ibrutinib-treated groups. **c** Representative pictures of lung sections with H & E stain taken at ×200 magnification. **d** Spleen weights were represented in grams from vehicle- and ibrutinib-treated mice. Gene expression analysis of **e** *Vegf*, **f** *Mmp9*, and **g** *Cxcl1* from the tumours of vehicle- and ibrutinib-treated mice. Data represented are means ± SEM from one of the three successful experiments with a minimum of five tumour samples per each group. *P < 0.05 and **P < 0.01, by unpaired *t* test.

Since tumour metastasis is an important determinant for the disease’s outcome and the survival of breast cancer patients, we analysed the effect of ibrutinib treatment on tumour metastasis to the lungs. We found that lungs from mice treated with ibrutinib contained significantly fewer lung metastatic nodules (both large and small) compared to the vehicle-treated group (Fig. 2a–c). In addition, ibrutinib-treated mice displayed less splenomegaly compared to the vehicle-treated mice (Fig. 2d). Next, we analysed the expression of genes such as *Vegf*, *Mmp9*, and *Cxcl1*, which are known to play an important role in tumour progression and metastasis. We found that tumours of ibrutinib-treated mice expressed significantly lower transcripts of *Vegf* (Fig. 2e), *Mmp9* (Fig. 2f), and *Cxcl1* (Fig. 2g) compared to tumours of the vehicle-treated group. Taken together, our results demonstrate that ibrutinib treatment reduces breast cancer metastasis and disease progression and is associated with a reduction in expression of tumour-promoting host factors.

### Ibrutinib treatment reduces the accumulation of MDSCs in tumour-bearing mice

**Fig. 3:**
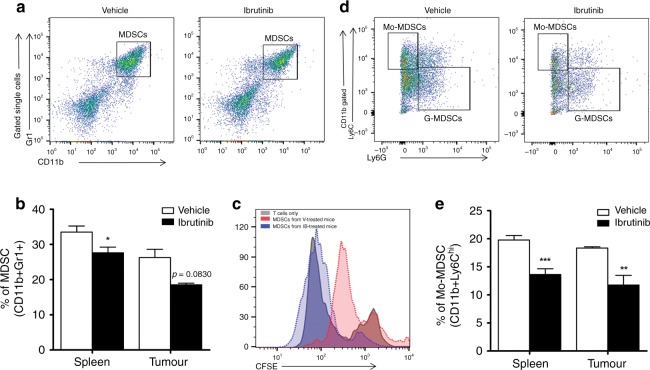
Reduced frequencies of total MDSCs and Mo-MDSCs by ibrutinib treatment. Single-cell suspensions were prepared from both groups of mice and analysed for total MDSCs (CD11b+Gr1+), monocytic MDSCs (Mo-MDSCs/CD11b+Ly6C^hi^), and granulocytic MDSCs (G-MDSCs/CD11b+Ly6G+) in both the spleens and tumours. **a** Representative gating strategy used to analyse the total MDSCs in both the spleen and tumour. **b** Bar graph represents the percentage of total MDSCs in total gated live cells from the spleen and tumour of vehicle- and ibrutinib-treated groups. **c** The proliferation of CFSE-labelled naive T cells co-cultured with CD11b+Gr1+ cells sorted from the spleens of vehicle- and ibrutinib-treated mice. **d** Represents the gating strategy followed to analyse the Mo-MDSCs and G-MDSCs from total CD11b+ gated live splenocytes. **e** Percentage of Mo-MDSCs from total CD11b+ gated live cells in the spleens and tumours of vehicle- and ibrutinib-treated mice. Data are representative of 1 of the 3 independent experiments, with 10–12 mice/group. Error bars represent means ± SEM. *P < 0.05, **P < 0.01, and ***P < 0.0001, by unpaired *t* test.

Many recent studies have shown the key role of MDSCs in tumour progression and in dampening antitumour immune responses.^19,20^ A recent study found that MDSCs express BTK, and treatment with ibrutinib reduces MDSC migration and frequency in vivo during experimental breast carcinogenesis.^6^ In this context, we analysed the frequencies of MDSCs (CD11b+Gr1+) in the spleens and tumours of vehicle- and ibrutinib-treated mice. Our data revealed that ibrutinib-treated mice contained significantly fewer MDSCs in the spleens and also a lower proportion of MDSCs in the tumours (Fig. 3a, b) compared to their counterparts. To confirm the identity of CD11b+Gr1+ cells as MDSCs, we performed an in vitro MDSC–T cell co-culture proliferation assay. CD11b+Gr1+ cells were isolated from the spleens of vehicle- and ibrutinib-treated tumour-bearing mice and plated with CFSE (carboxyfluorescein succinimidyl ester)-labelled T cells obtained from naive WT BL/6 mice. After stimulation with anti-mouse CD3 for 72 h, we analysed T cell proliferation through flow cytometry. Our results show that T cells co-cultured with MDSCs from ibrutinib-treated mice are able to proliferate significantly more compared to the T cells co-cultured with MDSCs from the vehicle-treated group (Fig. 3c). In addition to this, tumours of ibrutinib-treated mice expressed significantly lower transcripts of *Ccl2*, which is a key regulator of monocytes and MDSC recruitment to the tumour site.^20–22^

MDSCs are known to be a heterogeneous cell population based on the expression of CD11b, Ly6C, and Ly6G: Monocytic MDSCs (Mo-MDSCs; CD11b+Ly6C^hi^ Ly6G−) and granulocytic MDSCs (G-MDSCs; CD11b+Ly6C^low^ Ly6G+).^18,23,24^ It has been shown that Mo-MDSCs from metastatic breast cancer patients are immunosuppressive and the proportion of Mo-MDSCs correlates with breast cancer progression and metastasis in human breast cancer patients.^25^ In the present study, we found that ibrutinib treatment significantly reduced the Mo-MDSCs in the spleens and tumours (Fig. 3d, e). No significant difference in the number of G-MDSCs was observed between the groups (Fig. 3d). Collectively, these findings suggest that ibrutinib treatment reduces the MDSC populations in tumour-bearing mice, which could potentially contribute to lower tumour burdens in these animals.

### Ibrutinib increases the frequency of mature DCs in tumour-bearing mice by switching MDSCs to mature DCs

**Fig. 4:**
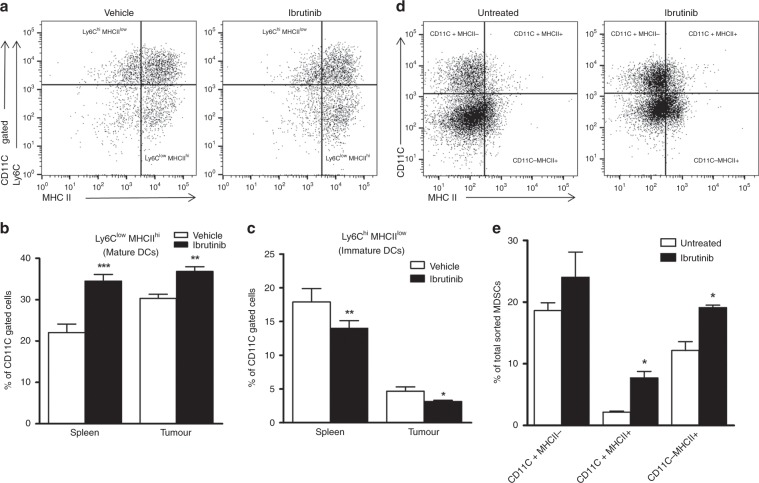
Ibrutinib switches MDSCs to mature dendritic cells. Single-cell suspensions were prepared from both the spleens and tumours of both groups of mice and analysed for mature DCs (Ly6C^low^ MHCII^hi^) and immature DCs (Ly6C^hi^ MHCII^low^) cells. **a** Representative gating strategy followed to analyse the mature and immature DCs from total CD11C gated live splenocytes. Bar graphs represent the percentage of **b** mature DCs and **c** immature DCs from total gated CD11C+ live cells from the spleens and tumours of vehicle- and ibrutinib-treated mice. **d**, **e** Total MDSCs were sorted from the spleens of WT tumour-implanted mice and treated with DMSO or ibrutinib (1 µM) for 1 h. Cells were washed, stained with CD11C and MHCII, and analysed for DC maturation by flow cytometry. **d** Gating strategy followed to identify the mature DCs (CD11C+MHCII+) from the total DMSO/ibrutinib-treated MDSCs. **e** The bar graph represents the percentage of matured DCs from total DMSO/ibrutinib-treated MDSCs. Data are representative of 1 of the 3 independent experiments, with 10–12 mice/group. Error bars represent means ± SEM. *P < 0.05, **P < 0.01, and ***P < 0.0001, by unpaired *t* test.

It is well documented that DCs play a major role in immunity against cancer and the ability of DCs to induce antitumour immunity depends on their maturation.^26,27^ Earlier studies show that murine DCs express Btk and that Btk-deficient DCs display a more mature phenotype and express higher levels of MHCII as well as co-stimulatory molecules.^15,28^ Previous studies have also shown that maturation of DCs is characterised by the downregulation of Ly6C and upregulation of MHCII and CD80.^16,29,30^ Hence, we analysed the mature (CD11C+Ly6C^low^ MHCII^hi^) and immature (CD11C+Ly6C^hi^ MHCII^low^) DC populations in the spleens of ibrutinib- and vehicle-treated mice. We found that ibrutinib-treated mice have significantly higher numbers of mature DCs in both the spleens and tumours compared to the vehicle-treated mice (Fig. 4a, b). Further, Ibrutinib treatment reduced the numbers of immature DCs in the spleens and tumours (Fig. 4a, c). No significant difference was observed in CD11C+Ly6C+MHCII+ cells between the groups. As the spleens and tumours of ibrutinib-treated mice contained higher proportions of mature DCs, we explored whether or not ibrutinib can promote the development of DCs from MDSCs, which are known to express BTK and hence could be targeted by ibrutinib. We isolated the MDSCs from tumour-bearing mice and treated them with 1 µM ibrutinib/DMSO for 1 h ex vivo. We found that MDSCs treated with ibrutinib showed enhanced expression of CD11C and MHCII compared to MDSCs treated with DMSO (Fig. 4d, e). Not only did ibrutinib significantly increase mature DCs (CD11C+MHCII+) from MDSCs but it also significantly increased the number of MHCII-expressing MDSCs (Fig. 4e). Together, these data suggest that ibrutinib mediates its antitumour activity at least in part by promoting the development of DCs from MDSCs.

### Ibrutinib treatment promotes T cell effector functions in tumour-bearing mice

**Fig. 5:**
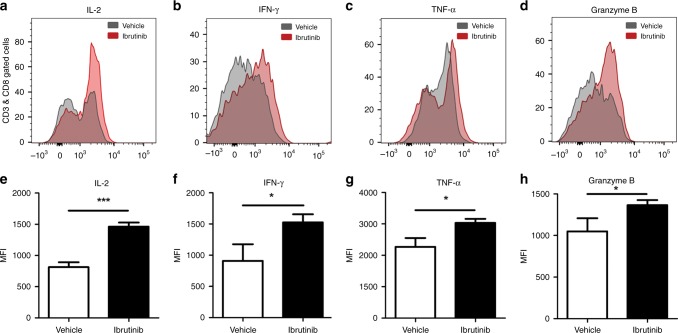
Ibrutinib enhances T cell proliferation and effector function of CTLs in vivo. Splenocytes were harvested from the vehicle and ibrutinib-treated mice; stained with CD3, CD4, and CD8; and analysed for IL-2, IFN-γ, TNF-α, and Granzyme B production by intracellular flow cytometry. **a** Expression and **e** mean fluorescence intensity (MFI) of IL-2, **b** expression and **f** MFI of IFN-γ, **c** expression and **g** MFI of TNF-α, **d** expression and **h** MFI of Granzyme B of CD8+ T cells from splenocytes of vehicle- and ibrutinib-treated mice. Error bars represent means ± SEM. Data are representative of 1 of the 3 independent experiments, with 10–12 mice/group. *P < 0.05, and ***P < 0.0001, by unpaired *t* test.

CTLs recognise antigenic peptides on tumour cells and elicit tumouricidal functions.^31,32^ However, some studies have shown that CTLs fail to produce inflammatory cytokines that promote tumour cell death.^33,34^ Ibrutinib has been shown to promote both CD8+ and CD4+ T cells by driving T_H_1-selective pressure in T lymphocytes.^1,35^ We therefore determined the effect of ibrutinib treatment on proliferation and effector functions of T cells in vivo. Ibrutinib treatment significantly enhanced the production of IL-2 (Fig. 5a, e), IFN-γ (Fig. 5b, f), and TNF-α (Fig. 5c, g) by CD8+ T cells compared to the vehicle treatment. In addition, CD8+ T cells of ibrutinib-treated mice displayed increased production of Granzyme B (Fig. 5d, h), which is perhaps not surprising as activated CD8+ T cells produce Granzyme B^36^ to suppress metastasis in breast and lung cancers.^37^ We also found that CD4+ T cells from ibrutinib-treated mice produce more IL-2 and IFN-γ compared to controls, but the differences were statistically not significant (data are not shown). Taken together, these results indicate that ibrutinib treatment in mice with breast cancer enhances T cell effector functions.

### Ibrutinib enhances T cell proliferation and promotes T_H_1 cytokines

**Fig. 6:**
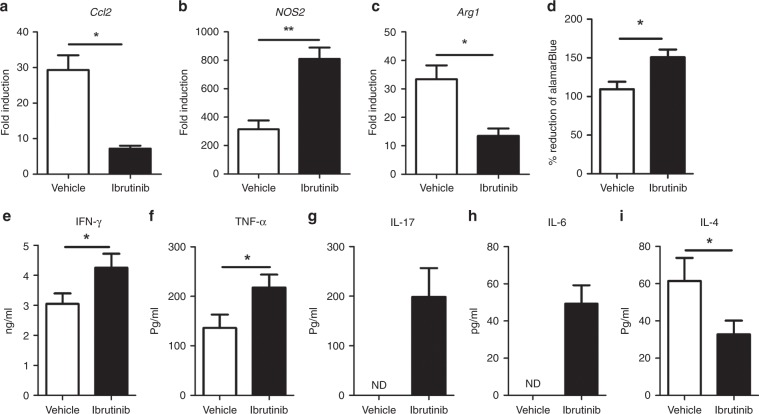
Increased production of T_H_1 cytokines by splenocytes of ibrutinib-treated mice. Mice were euthanised at day 30, and tumours and spleens were collected from both vehicle- and ibrutinib-treated mice. Gene expression analysis of **a** *CCL2*, **b** *NOS2*, and **c** *Arginase1* from tumours. Splenocytes were stimulated with 2 µg/ml of anti-mouse CD3/CD28 for 72 h and analysed for T cell proliferation and cytokine production. **d** T cell proliferation analysed by the Alamar Blue reduction method. **e** IFN- γ, **f** TNF-α, **g** IL-17, **h** IL-6, and **i** IL-4, and cytokine production quantified by ELISA. Data are representative of 1 of the 3 independent experiments, with 10–12 mice/group. Error bars represent means ± SEM. *P < 0.05 and **P < 0.01, by unpaired *t* test.

Since CD8+ T cells in the spleens of ibrutinib-treated mice displayed enhanced production of IL-2 and other T_H_1 cytokines, we further analysed the production of T_H_1 and T_H_2 cytokines from anti-CD3/CD28-stimulated splenocytes of ibrutinib- versus vehicle-treated mice. Consistent with the results observed by intracellular staining, we found that splenocytes from ibrutinib-treated mice have shown increased T cell proliferation (Fig. 6d). Also, splenocytes from ibrutinib-treated mice showed significantly higher amounts of IFN-γ (Fig. 6e), TNF-α (Fig. 6f), IL-17 (Fig. 6g), and IL-6 (Fig. 6h), while a decrease in T_H_2-associated IL-4 production was observed (Fig. 6i). Furthermore, ibrutinib treatment significantly enhanced *iNOS* (Fig. 6b) and diminished *Arginase1* (Fig. 6c) expression in the tumour, which is perhaps not surprising because ibrutinib is known to induce T_H_1 selective pressure on T lymphocytes resulting in the production of higher IFN-γ and inducible nitric oxide synthase (iNOS).^1^ Taken together, these results indicate that ibrutinib enhances T cell proliferation and T_H_1 effector response.

## Discussion

Originally recognised for its role in B cell signalling, BTK has emerged as an essential regulator of immune responses and is redundantly expressed by many hematopoietic cells, including myeloid cells. It is known to play an essential role in the maturation and function of myeloid cells.^4,6^ In this study, using a murine experimental model of breast cancer, we demonstrate that the potent BTK/ITK inhibitor ibrutinib is effective in inhibiting breast cancer tumour growth and metastasis. Although we show the direct effect of ibrutinib on E0.2 breast cancer cells, we also show that ibrutinib mediates its antitumour activity at least partly by acting on MDSCs and promoting their differentiation to mature DCs, which boosts antitumour T_H_1 and CTL immune responses. To the best of our knowledge, our study is the first report to demonstrate the direct effect of ibrutinib on MDSCs and promoting the generation of DCs from them.

Current cancer immunotherapies involve small-molecule checkpoint inhibitors or blocking antibodies. The antibody-blockade strategy has yielded success as a combination therapy; however, it possesses the risk for a variety of side effects that require supportive care services. Among small-molecule inhibitors, ibrutinib has been reported to promote the development of DCs in bone marrow cells, polarise immune responses to T_H_1, and exert antitumour immunomodulatory effects on immune cells.^1,38^

MDSCs are known to contribute to tumour progression,^12,13^ express BTK, and can differentiate into macrophages and DCs^39,40^ in the peripheral lymphoid organs. However, MDSCs recruited to the tumour display immunosuppressive properties due to inhibition and differentiation factors present in the tumour microenvironment. Given the role of MDSCs in cancer progression, kinase inhibitors that block MDSC generation have been found to be effective in cancer therapy.^41,42^ One such inhibitor is ibrutinib, which has been shown to reduce the frequency of MDSCs in breast cancer.^6,43^ In the present study, we hypothesised that ibrutinib reprogrammes MDSCs to mature DCs, which trigger T_H_1-mediated antitumour immunity. In line with the above studies, we found that ibrutinib treatment reduced MDSC populations but increased mature DCs in the spleen and tumours. Furthermore, treatment reduced Mo-MDSCs, which are known to play a significant role in tumour progression and metastasis.^25^ These results suggest that ibrutinib treatment significantly reduced the migration of tumour-promoting myeloid cells to the secondary sites and resulting in diminished metastasis. Ibrutinib treatment also significantly reduced the expression of *Vegf*, *Mmm9*, and *Cxcl1*, which are known to play a major role in tumorigenesis, metastasis, and angiogenesis.

It is well recognised that immature DCs might exhibit immunosuppressive and/or tolerogenic effects,^29,44^ and their maturation depends on the local microenvironment. It has been shown that BTK negatively regulates maturation of DCs and BTK^−/−^ DCs exhibit more mature phenotypes and stronger T cell-stimulatory ability.^15^ A recent study from our group identified that ibrutinib-treatment-induced DC activation and maturation by upregulating CD80, MHC-II, and C-C chemokine receptor type 7.^16^ In line with these findings, our present study revealed that ibrutinib treatment significantly increased mature DCs in the spleens as well as tumours while the immature DC proportion remained elevated in the vehicle group. We also found that ex vivo treatment of MDSCs with ibrutinib significantly enhanced the expression of CD11C and MHCII molecules, which indicates a change in their phenotype to mature DCs. Together, these findings indicate that Ibrutinib mediates its anticancer activity at least in part by promoting the conversion of MDSCs into mature CD11C+MHCII+ DCs. Furthermore, our findings suggest that the BTK pathway negatively regulates the conversion of MDSCs into mature DCs.

Previous studies have found that ibrutinib markedly improves T cell numbers and function in CLL patients.^35,45^ Furthermore, ibrutinib has been shown to increase the production of pro-inflammatory cytokines IFN-γ, IL-6, and TNF-α and concomitantly suppress the production of anti-inflammatory cytokines IL-4, IL-13, and IL-10.^1,46^ Consistent with these findings, we also found that the splenocytes of ibrutinib-treated mice showed enhanced proliferation and skewed T_H_1 responses through elevated levels of IFN-γ, IL-6, and TNF-α. It has been also shown that T_H_17 responses alleviate cancer pathogenesis and improve survival in CLL cases.^47,48^ We previously reported that ibrutinib enhances IL-17 responses, which modulate antigen presentation and DC functions.^17^ Similarly, in our present study, we found that ibrutinib treatment was associated with a significant increase in IL-17 production, which could be responsible for enhancing T cell-mediated antitumour responses.

In recent years, DC-based cancer immunotherapeutic strategies have focused on increasing DC recruitment to the tumour microenvironment. However, the recruitment of progenitor DCs to the tumour site may not be sufficient for eliciting antitumour immunity since the immunosuppressive environment redirects their development into MDSCs. On the contrary, the recruitment of mature DCs could significantly potentiate CTL responses and overcome the MDSC-based barrier for cancer immunotherapy. MDSC-induced T cell dysregulation and inhibition of CTL responses remain a common feature in cancer pathogenesis.^49^ This immune dysregulation of CD8+ T cells and impaired CTL effector responses can be attributed to prevailing immunosuppressive conditions in the tumour microenvironment.^50,51^ In the present study, we found that ibrutinib treatment was associated with a significant enhancement of CTL activity as evident by significantly elevated levels of Granzyme B, IL-2, IFN-γ, and TNF-α production. These findings were consistent with previously reported in vivo studies where ITK^−/−^ T cells exhibited an increased frequency of activated T cells and prolonged survival.^52,53^

In conclusion, our results show that ibrutinib treatment is effective in the suppression of breast cancer tumour progression and metastasis. The BTK/ITK inhibitor ibrutinib reprogrammes the tumour-induced immunosuppressive population of myeloid lineage (MDSCs) into mature DCs by blocking BTK, increases the proportion of mature DCs in the tumour and lymphoid organs, and promotes antitumour T cell activity. Together, our findings indicate that ibrutinib and other BTK inhibitors could be novel drugs for the treatment of breast cancer.

## Data Availability

All the data generated in the present study are included in this article. The data presented in this article will be available from the corresponding author upon request.
